# GREATER PHYSICAL ACTIVITY AND SOCIAL ENGAGEMENT OFFER PROTECTION AGAINST COGNITIVE DECLINE OVER TIME

**DOI:** 10.1093/geroni/igad104.3375

**Published:** 2023-12-21

**Authors:** Usha Dhakal, Sara McLaughlin, Seonjin Kim, Amy Roberts, Jonathon Vivoda, J Scott Brown

**Affiliations:** Miami University, Oxford, Ohio, United States; Miami University, Oxford, Ohio, United States; Miami University, Oxford, Ohio, United States; Miami University, Oxford, Ohio, United States; Miami University, Oxford, Ohio, United States; Miami University, Oxford, Ohio, United States

## Abstract

Evidence suggests that physical activity and social engagement in later life are associated with a less pronounced and reduced risk of cognitive decline over time; however, findings are mixed and knowledge concerning the effect of these factors on cognitive functioning in older adults with mild cognitive impairment (MCI) is underdeveloped. To enhance understanding, we extracted data from eight waves (2004-2018) of the Health and Retirement Study and used hierarchical linear mixed models to investigate the impact of physical activity and social engagement on cognitive functioning in Americans aged 60 years and older with MCI (n=1462). The average cognitive score (sum of immediate and delayed word recall, serial seven subtraction test, counting backwards, range: 0 to 27) at baseline was 9.6, which decreased by 0.14 points each year. Each unit increase in physical activity (weighted sum of light, moderate, vigorous activity; range: 9 to 45) and social engagement (measure capturing social visits, volunteerism, religious participation; range: 0 to 3) reduced the effect of time on cognitive functioning by 0.01 (β=0.007, p<.0001) and 0.04 (β=0.040, p<.0001) points, respectively, while simultaneously controlling for APOE genotype and other covariates. This means, for example, that the cognitive score of someone with a physical activity score of 40 was, on average, 3.1 points higher than someone with a physical activity score of 10 after 16 years of follow-up. Findings suggest that greater physical activity and social engagement offer protection against cognitive decline and could minimize MCI progression among older adults experiencing MCI.

